# *Armigeres subalbatus* incriminated as a vector of zoonotic *Brugia pahangi* filariasis in suburban Kuala Lumpur, Peninsular Malaysia

**DOI:** 10.1186/1756-3305-6-219

**Published:** 2013-07-30

**Authors:** Azdayanti Muslim, Mun-Yik Fong, Rohela Mahmud, Yee-Ling Lau, Sinnadurai Sivanandam

**Affiliations:** 1 Faculty of Medicine, Universiti Teknologi MARA (UiTM), Sungai Buloh, Selangor, Malaysia; 2 Department of Parasitology, Faculty of Medicine, University of Malaya, 50603 Kuala Lumpur, Malaysia; 3 Tropical Infectious Diseases Research and Education Centre (TIDREC), Faculty of Medicine, University of Malaya, 50603 Kuala Lumpur, Malaysia

**Keywords:** *Armigeres subalbatus*, Vector, Zoonotic, *Brugia pahangi*, Filariasis

## Abstract

**Background:**

In 2011, we reported occurrence of natural human infections with *Brugia pahangi*, a filarial worm of dogs and cats, in a surburb of Kuala Lumpur, the capital city of Malaysia. Our preliminary entomological survey at that time suggested the mosquito species *Armigeres subalbatus* as the vector of the zoonotic infections. In this present report, we provide biological evidence to confirm our preliminary finding.

**Findings:**

A total of 1798 adult female *Ar. subalbatus* mosquitoes was caught in the vicinity of the suburb, and 1599 were dissected for the presence of filarial larvae. Sixty-two mosquitoes were positive, and 27 of these were infected with L_3_ larvae_._ The L_3_ were inoculated into male gerbils. Microfilariae could be detected in the gerbils 92 days post-infection. Post-mortem on the gerbils recovered adult worms in the peritoneal cavity, heart, lungs, tail and testis. Male adult worms were confirmed to be *B. pahangi* by the ratio length of their spicules (left spicule: right spicule). Female adult worms were confirmed by the absence of minute cuticular bosses in the tail region. The worms were further confirmed to be *B. pahangi* by PCR.

**Conclusions:**

Our results showed that *Ar. subalbatus* was the vector for the zoonotic *Brugia pahangi* infections. This mosquito species should now be categorised as a medically important mosquito species in Malaysia. Its role in the transmission of zoonotic *B. pahangi* must therefore be considered in future studies on filarial infections.

## Findings

### Background

*Brugia pahangi*, a closely related species of *B*. *malayi*, is a lymphatic filarial worm of mammals, but essentially of domestic cats and dogs [1]. This worm is not known to cause human disease in the natural environment. Although there was a report on the presence of its microfilariae (mf) in human blood in South Kalimantan, the finding was not conclusive as the method used to identify the mf relied on measuring phosphatase distribution in the mf [2]. Experimental *B*. *pahangi* infection on human volunteers produced signs and symptoms of lymphatic filariasis, but hardly produced microfilaraemia [3].

Recently, we reported several cases of natural human infection with *B. pahangi* in a suburb of Kuala Lumpur, the capital city of Malaysia [4]. The life cycle of *B. pahangi* involves an intermediate mosquito vector and primary mammalian hosts. Mosquito species *Mansonia annulata* and *Ma. dives* are known to be natural vectors of *B. pahangi*[5]. However, these two species live in a forest environment, and they were not found in our preliminary entomological survey of the suburb. Instead, the most prevalent mosquito species in the suburb was *Armigeres subalbatus* and some were found to be infected with filarial larvae.

*Ar. subalbatus* is commonly found close to human dwellings especially in sub-urban areas with poor sanitation that contain polluted water such as septic tanks [6]. This species has been known to be a vector of Japanese encephalitis virus [7]. It has also been reported to be a vector of filarial worm *Wuchereria bancrofti* in India [8] and the dog heartworm *Dirofilaria immitis* in Peninsular Malaysia [9]. In the early 1960s, it was found that *Ar. subalbatus* could serve as an efficient vector in experimental infections involving *B. pahangi*. Since then, numerous *B. pahangi*-*Ar. subalbatus* research work has been conducted. However, despite being widely used as a laboratory vector for several decades, hitherto there is no detailed description of *Ar*. *subalbatus* as a vector for *B*. *pahangi* in nature. In this report, we present biological findings to confirm *Ar*. *subalbatus* as a natural vector, particularly for the transmission of zoonotic *B*. *pahangi* filariasis.

## Methods

### Location of entomological survey

Entomological survey was carried out in the vicinity of Bukit Gasing–Kampung Kerinchi (3°5′47″N, 101°39′25″ E – 3°6′51″N, 101°39′47″E), where zoonotic *B. pahangi* infections were previously reported [4]. This suburb consisted of houses, apartments, several construction projects and slum villages. A recreational secondary forest straddles the center of the suburb. Originally, the secondary forest was a plantation site and had been left idle for 50 years.

### Mosquito collection method

Mosquito collection was carried out from 0600–0900 hours and from 1800–2030 hours using the human landing catches method. Approval for the collection was obtained from the University of Malaya Ethical and Research Review Committee [Ref. No. PAR/19/02/2013/AA (R)]. The mosquitoes were brought alive to the laboratory in the Department of Parasitology, Faculty of Medicine, University of Malaya to be counted and identified.

### Dissection of mosquitoes

Dissection of mosquitoes was performed at the earliest possible time to check for the presence of filarial larvae. Only female mosquitoes were dissected. The mosquito was placed on a slide under a binocular dissecting microscope. The wings and legs were removed and the body separated into three major parts: head with proboscis, thorax and abdomen. Each part was placed in a separate drop of saline. Live larvae could be seen wriggling out into the normal saline. The number of infected mosquitoes, and the number and the stage of the larvae obtained were recorded.

### Inoculation of stage 3 larvae (L_3_) into gerbils (*Meriones unguiculatus*)

The L_3_ larvae were pooled and inoculated immediately into male gerbils by intra-peritoneal and subcutaneous injections for development into adult worms. Sluggish and inactive larvae were not used. After 3 months, thick blood smears of the gerbils were made and stained with Giemsa for mf detection [10]. Approval for using gerbils in our study was granted by the University of Malaya Animal Care and Use Committee [Ref. No. PAR/29/06/2012/RM(R)].

### Post mortem and recovery of adult worms

The gerbils were sacrificed for recovery of adult worms 95 days post-infection. A midline incision was made with iris scissors. After skinning and opening up the abdominal cavity, the testis, lungs, heart, lymph nodes and tail tissues were removed and placed in separate small petri dishes containing normal saline. The adult worms were then isolated from the tissues using a dissecting needle and transferred to a cavity block containing normal saline [11].

### Identification of adult worm species by morphometric method

The adult worm was mounted in glycerin in a permanent hanging drop preparation on a slide. It was then examined under the microscope which was attached with an Image Analyzer. The length of the left spicule (LS) and right spicule (RS) of male worms was measured and the LS:RS ratio was calculated. The ratio range for *B. pahangi* is 1.80-2.50:1, while for *B. malayi* is 2.90-3.80:1 [12]. The tail region of female worms was also examined to determine the presence of minute cuticular bosses, a characteristic of adult female worm of *B. malayi*, but absent in *B*. *pahangi*.

### Identification of adult worm species by PCR

DNA was extracted from adult worms using DNeasy Blood and Tissue Kit (Spin-Column protocol, QIAGEN, Germany). The extracted DNA was amplified using primer pairs specific for the cytochrome oxidase I (*COX*I) gene of *B*. *pahangi* (forward primer 5′ TATTGCCTGTTATGC 3′, reverse primer 5′ TGTATATGTGATGAC 3′). PCR was carried out in a 25 ml reaction mixture containing 10 mM Tris–HCl (pH 8·3), 2 mM MgCl_2_, 50 mM KCl, 0·01% gelatin, 200 mM of each deoxynucleoside triphosphate, 20 pmol of each primer, 1 U of Taq polymerase (Fermentas Life Sciences, Canada). The PCR mixture was pre-heated at 95°C for 10 min for initial denaturation before 30 cycles of amplification, which consisted of denaturation at 94°C for 1 min, annealing at 54°C for 1 min, and elongation at 72°C for 2 min. Final extension of the reaction was carried out at 72°C for 10 min. PCR product was analysed by agarose gel electrophoresis. The expected size of the PCR product was 633 bp.

## Results

### Mosquito collection

In the entomological survey, a total of 1878 mosquitoes was collected (Table 1). Four species of mosquitoes from two genera were found during the collection period. The predominant mosquitoes caught were *Ar. subalbatus* (95.7%). Another *Armigeres* species, *Ar. kesseli*, was also caught, albeit in small number. Other mosquitoes collected were *Aedes albopictus* and *Ae. aegypti.*

**Table 1:** Mosquito species and number collected in the entomological survey

**Mosquito species**	**Number of mosquitoes collected (%)**
*Armigeres subalbatus*	1798 (95.7)
*Armigeres kesseli*	16 (0.9)
*Aedes albopictus*	55 (2.9)
*Aedes aegypti*	9 (0.5)
**Total**	**1878**

### Mosquito dissection and larvae recovery

Of the 1798 *Ar. subalbatus* mosquitoes collected, 1599 were dissected for the presence of filarial larvae. Sixty-two mosquitoes were positive, and 27 of these were infected with L_3._ Majority of the infected mosquitoes harboured between 7 and 25 L_3,_ although there was one which had 45 L_3_. Most of the L_3_ were found in the proboscis. However, in some mosquitoes which had a high number of larvae, L_3_ could be found in the entire body. Developing larvae (L_1_ and L_2_) were found mostly in the thorax. The total larvae recovered were 346 L_3_, 259 L_2_ and 16 L_1_. Table 2 summarises the number of positive mosquitoes, infection and infective rates, and number of larvae recovered. None of the *Ar. kesseli*, *Ae. albopictus* and *Ae. aegypti* mosquitoes were positive for larvae.

**Table 2:** Number of positive mosquitoes, infection and infective rates, and number of larvae recovered

**Infection/infective rate**	**Number of infected mosquitoes (%cih)**
Total positive (infection rate)	62 (3.9)
with L_3_ (infective rate)	27 (1.7)
with L_2_	32 (2.0)
with L_1_	3 (0.2)
**Larva stage**	**Total larvae recovered in mosquitoes**
L_3_	346
L_2_	259
L_1_	16

### Pre-patent period, recovery of adult worms and species identification

Only L_3_ larvae were injected into two gerbils. Thirty-four were inoculated intra-peritoneally into the first gerbil, and 5 subcutaneously into the second gerbil. Blood from the infected gerbils was taken at 1000 hours and examined for the presence of mf for six days, from day 90–95 post infection. The blood smear was stained with Giemsa. Mf could be detected on days 92–95, indicating pre-patent period in gerbil of 92 days.

**Figure 1:**
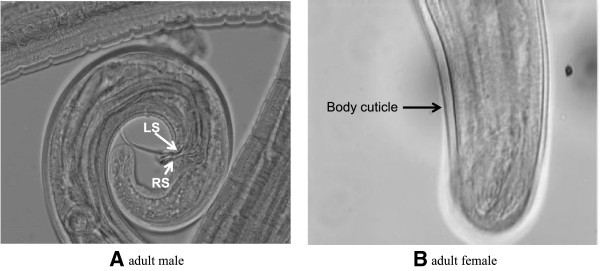
**Identification of species based on: (A) the ratio length of spicules (left spicule:right spicule, LS:RS) in adult male, the worm was confirmed to be *B. pahangi *with LS:RS = 2.00:1; and (B) tail region of adult female of *B. pahangi*, with body cuticle devoid of minute cuticular bosses (x40).**

Post mortem on the gerbils was carried out 95 days post-infection. Eighteen adult worms were recovered, in which 16 were recovered in the peritoneal cavity, heart, lungs and tail of the first gerbil. Two were recovered from the testis of the second gerbil. Five of the 7 adult male worms were cleared and mounted for identification. They were confirmed to be *B. pahangi* by determining the ratio length of their spicules (LS:RS). The ratios of the adult male worms obtained were 2.47:1, 2.36:1, 2.38:1, 2.06:1 and 2.00:1 (Figure 1A), respectively. These ratios were within the ratio range for *B. pahangi* (1.80-2.50:1**)** which is much smaller than the ratio of *B. malayi* (2.90-3.80:1) [12]. The 9 adult female worms were confirmed to be *B. pahangi* by examining their body cuticle in the tail region. The body cuticle of *B. pahangi* adult female worm is devoid of the minute cuticular bosses (Figure 1B), which are only seen in female *B. malayi* adult worm [12].

### Species identification of adult worm by PCR

**Figure 2:**
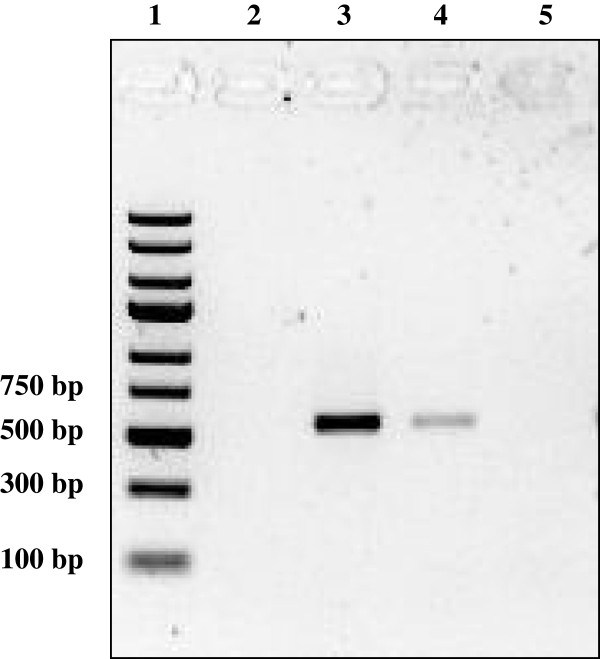
**Agarose gel electrophoresis of amplicons from PCR using primers specific for *Brugia pahangi COX*I gene.** Lane 1, DNA molecular mass standards (Fermentas, Lithuania); lane 3, amplicon (633 bp) from PCR on DNA of adult male worm; lane 4, amplicon (633 bp) from PCR on DNA of adult female worm; no amplicon was detected from PCR on DNA of *B. malayi* adult male worm (lane 2) and negative control (water only) (lane 5).

The adult worms were further confirmed to be *B. pahangi* by PCR using primers specific for the *COX*I gene. The 633 bp region of the *B. pahangi COX*I gene was successfully amplified (Figure 2).

## Discussion

*Armigeres subalbatus* is widely distributed in Malaysia. Previously, this species has never been considered a medically important mosquito in Malaysia as compared to other mosquitoes such as *Culex*, *Aedes*, *Mansonia* and *Anopheles* spp. Our present study highlights the role of *Ar. subalbatus* as a vector of zoonotic filariasis in Peninsular Malaysia.

Pioneering work on the vectors of *B*. *pahangi* in Malaysia was carried out mainly in the 1960s. Field and laboratory studies established *Ma. annulata* and *Ma. dives* as vectors of *B*. *pahangi* in nature [5]. The typical breeding place for *Ma*. *annulata* is the forest verge where open swamp and forest meet. Swamp forest having rootlets of trees, rattans and palms is the usual breeding ground for *Ma. dives*. Our study site, on the other hand, was a suburb with residential houses and flats, villages and a recreation hill park. This therefore explains the absence of *Mansonia* species in the site. The surroundings of the site were favourable for *Ar. subalbatus*, which breeds well around human dwellings with poor sanitation that contain polluted water [6]. This explains the abundance and predominance of *Ar. subalbatus* in the site as compared to other mosquito species, such as *Ar. kesseli*, *Ae. albopictus* and *Ae. aegypti.* The human landing catch method was used to collect the mosquitoes, and the high number of *Ar. subalbatus* obtained was surprising since it was previously reported that this species did not feed readily on humans [13]. Infection and infective rates obtained in our study were 3.9% and 1.7%, respectively. These rates are higher than those observed in previous entomological studies in endemic areas in Malaysia, which ranged from 0.1% to 3% [14-17].

A unique biological feature of *Ar. subalbatus* is its high susceptibility to *B. pahangi* but refractoriness to *B. malayi* mf infection. The mf of these filarial species are morphologically similar, although genetically they can be differentiated by various molecular methods. Whereas *B. pahangi* mf can easily develop to the infective L_3_ stage, *B. malayi* mf are rapidly destroyed in the haemocoel by melanotic encapsulation [18,19]. Before the advent of biochemical and molecular methods, distinguishing of *B. malayi* and *B. pahangi* mf could only be done by letting *Ar*. *subalbatus* feed on the infected animal or human, and detect the development of larvae in the mosquito [20]. Hence, the developing and infective larvae recovered in the *Ar. subalbatu*s in our study were undoubtedly *B. pahangi*.

In laboratory experimental settings, *Ar*. *subalbatus* has been demonstrated to be an extremely efficient host, producing high number of L_3_ larvae [5]. In an experiment in which *Ar. subalbatus* was allowed to feed on a *B. pahangi* animal carrier with more than 1 mf/μl, all become infected and the larvae reached maturity as early as the 7^th^ day after feeding. Almost all (99%) larvae were L_3_ by the 11^th^ day. When fed on an animal carrier with 6.2 mf/μl, an average of 38.7 larvae per mosquito was obtained. Wharton [13] reported experimental infection index of 7.1 ± 1.1, for mf counts of 1 mf/μl. In other words, of an average of 7 L_3_ per mosquito could be produced. In our study, a relatively high number of L_3_ larvae was seen in the naturally-infected mosquitoes, ranging between 7 and 25, with one reaching up to 45. This high infective rate is likely due to the size of the female *Ar. subalbatus*, which is comparatively larger than any other mosquito species. In a single blood meal, a female *Ar. subalbatus* can take an average of 4.5 μl of blood [13].

Upon ingestion of an infective blood meal by the female *Ar. subalbatus*, the mf penetrate the midgut epithelium and migrate to the thoracic musculature of the mosquito. Here, the mf moult several times to develop into infective L_3_. The L_3_ migrate to the head region and finally to the proboscis of the mosquito. The L_3_ are then transmitted to a vertebrate host when the infected mosquito takes a blood meal. In our study, most of the larvae were seen in the usual sites in the mosquitoes: L_1_ and L_2_ in the thorax, and L_3_ in the proboscis. Oddly, in some highly infected mosquitoes, L_3_ could be found in the entire body. Zahedi *et al*. [21], who found developing larvae L_2_ in the head, commented that erratic migration of larvae to unusual sites might be the consequence of heavy infection.

In our survey, *Ar. kesseli* was found but in smaller number. It shares similar morphology with *Ar. subalbatus*. The latter, however, can be identified by the presence of white scaling in the hind femur which tapers and terminates before the knee, and the broad black band on sternite 3–6. The white scaling in the hind femur of *Ar. kesseli* is broad, and the black band on its sternite is narrower [22]. Despite having morphological similarities and occupying similar habitats, none of the *Ar. kesseli* in our study was found to be infected with larvae.

## Conclusions

Our study was conducted in a suburb where several cases of zoonotic *B*. *pahangi* infection were recently reported [4]. The findings of our study show that *Ar. subalbatus* was indeed the vector for the zoonotic infections. With a capacity to naturally harbour a high number of *B*. *pahangi* larvae and being a human biter which thrives in areas of human habitations, *Ar. subalbatus* should be considered a medically important mosquito species in Malaysia along with other mosquitoes such as *Aedes*, *Anopheles*, *Culex* and *Mansonia* spp. Its role in the transmission of zoonotic *B. pahangi* must be considered in future studies on filarial infections.

## Abbreviations

L: Larva; LS: Left spicule; RS: Right spicule.

## Competing interests

The authors declare that they have no competing interests.

## Authors’ contributions

AM, MYF, and RM designed the study. AZ and SS performed field collection, mosquito identification and gerbil infection. AZ and YLL performed molecular identification of the adult worms. AZ and MYF drafted the manuscript. All authors read and approved the final version of the manuscript.
